# A Case of Pancreatic Neuroendocrine Tumor With Extensive Skeletal Metastases Detected on 68Ga-DOTANOC PET-CT

**DOI:** 10.7759/cureus.43130

**Published:** 2023-08-08

**Authors:** Ritwik Wakankar

**Affiliations:** 1 Nuclear Medicine, Max Super Speciality Hospital, New Delhi, IND

**Keywords:** chronic diarrhea, skeletal metastases, pancreatic net, dotanoc pet-ct, neuroendocrine tumor

## Abstract

Neuroendocrine tumors (NETs) are a rather uncommon cause of chronic non-bloody diarrhea and are therefore often left undiagnosed for prolonged periods of time. In this case, a 46-year-old man was inappropriately treated with antibiotics for months by various doctors, and by the time the diagnosis of NET was made, the tumor had already metastasized to the entire skeleton. The patient refused surgery and was started on octreotide, which resolved his diarrhea.

## Introduction

Neuroendocrine tumors (NETs) are derived from neurosecretory cells located in various parts of the body. These are rare tumors and occur mostly in the lungs, appendix, small intestine, rectum, and pancreas. They can be functional (produce their own hormones) and non-functional (do not produce any hormones). The diagnosis and treatment of these tumors depend on the size, the extent of the tumor, its location, how aggressive it is, and whether it produces hormones or not. NETs are a rare cause of chronic diarrhea. Due to this, it becomes difficult to diagnose them on time in patients, and this can have severe life-threatening consequences for the patients [1]. In this case, a patient with chronic diarrhea and asthenia for almost a year with no success in establishing a diagnosis underwent a 68Ga-DOTANOC PET-CT to evaluate for a potential NET. The patient was eventually diagnosed as a pancreatic NET with extensive skeletal metastasis. A 68Ga-DOTANOC PET-CT helped to diagnose a NET without the need for an invasive biopsy and also helped to evaluate the extent of the disease.

## Case presentation

A 46-year-old man presented to the emergency room of the hospital with complaints of multiple episodes of diarrhea, generalized body weakness, malaise, and nausea. He denied having any emesis. His personal history was significant for coronary artery disease for which he was taking medication as prescribed by his cardiologist. He denied any history of smoking, alcohol, or substance abuse. His family history was unremarkable. He also stated that he had been having similar episodes of non-bloody diarrhea on and off for the last eight months, but every time it used to resolve on its own after taking a course of antibiotics.

Blood samples and arterial blood gas (ABG) analysis were ordered for him. The tests revealed a slightly reduced sodium level (132 mmol/L) and metabolic acidosis (pH: 7.1 and bicarbonate: 6 mEq/L). C-reactive peptide (CRP) and total leucocyte count were within normal limits. During the period of his stay in the hospital, a contrast-enhanced CT (CECT) scan was ordered, which revealed a small lesion (~2 x 2 cm) in the pancreatic head region.

Suspecting a potential malignancy, a gastrointestinal (GI) surgery referral was ordered, and the patient underwent an endoscopic ultrasound, which corroborated the CT findings. Later on, a fine needle aspiration cytology (FNAC) was done from the lesion, which revealed cells with round nuclei, granular cytoplasm, positive staining for chromogranin A (CgA), and staining negatively for CD56, revealing the diagnosis to be a pancreatic NET. Serum chromogranin levels were only marginally elevated but given the fact that the patient was taking proton pump inhibitors (PPI) for his gastric reflux, these values seemed less reliable.

**Figure 1:**
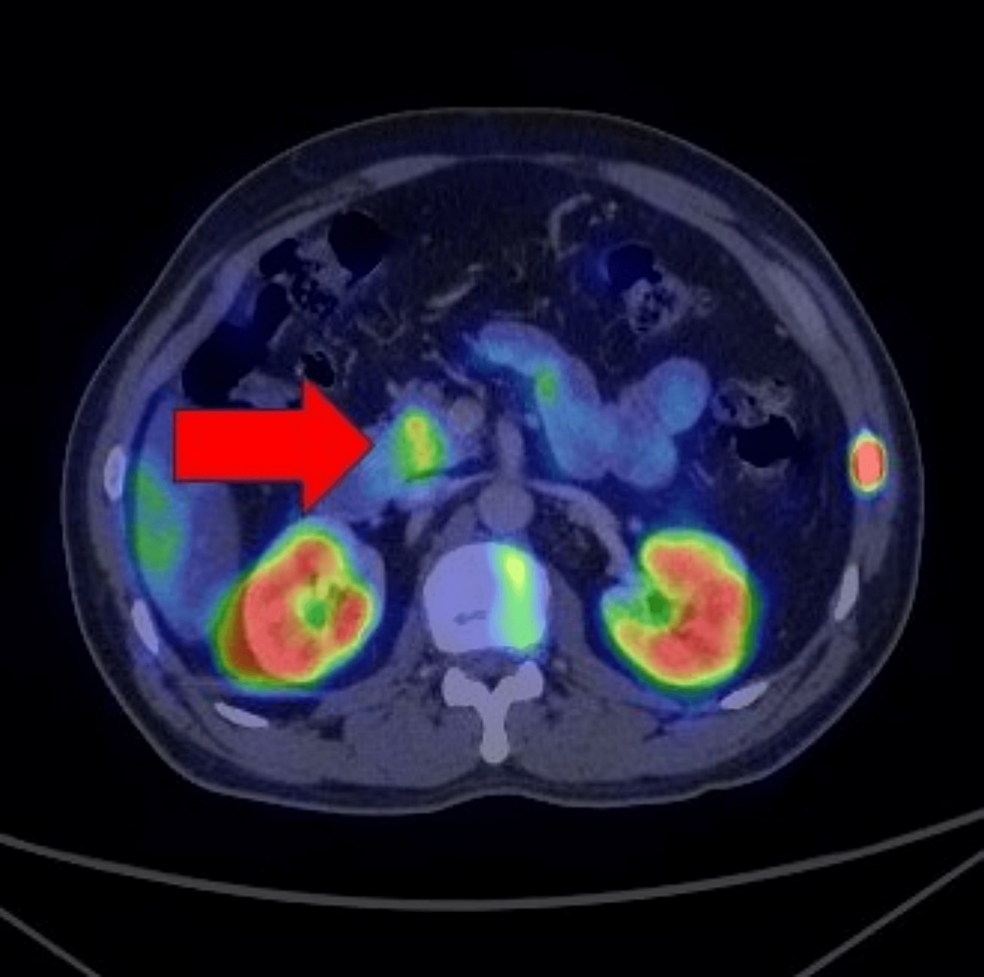
Axial 68Ga-DOTANOC PET-CT image showing the tracer avid pancreatic neuroendocrine tumor (red arrow).

**Figure 2:**
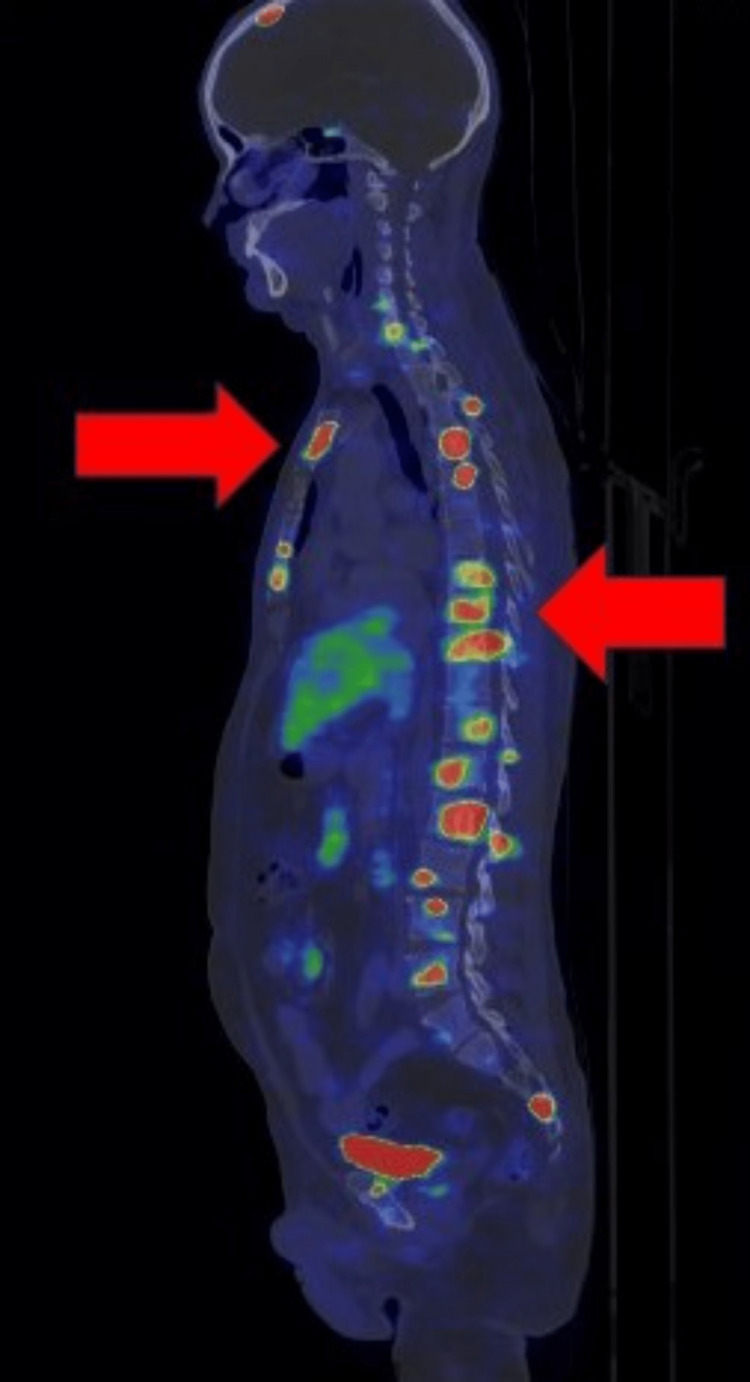
Sagittal 68Ga-DOTANOC PET-CT image showing tracer avid extensive skeletal metastases in the sternum (red arrow on the left) and multiple vertebrae (red arrow on the right).

A 68Ga-DOTANOC PET-CT was done to stage the disease and decide on the course of treatment. The scan revealed a DOTANOC-avid pancreatic head lesion and multiple DOTANOC-avid sclerotic metastatic skeletal lesions (Figures 1, 2). No lesions were noted in the liver or lungs. The patient refused to undergo any surgery and wished for non-invasive treatment. It was then decided to start him on 20 mg of octreotide, a long-acting release, following which his diarrhea resolved. He was advised to follow up in the outpatient department (OPD) after three months but ended up migrating to a different hospital and was thus lost to follow-up.

## Discussion

This case report is meant to highlight the role that 68Ga-DOTANOC PET-CT plays in the work-up of metastatic NET as well as the role of somatostatin analogs in the management of these hormonal symptoms in these patients [2,3]. NETs express somatostatin receptors (SSTRs) on their surfaces, and it is these receptors that are detected by 68Ga-DOTANOC PET-CT. By detecting them, not only can we evaluate the disease burden in NETs but also decide on which patients would be appropriate candidates for the use of somatostatin analogs. A 68Ga-DOTANOC PET-CT can also be used to assess whether or not the disease is responding to the therapy long before any morphological changes become obvious on CT imaging. The somatostatin analogs bind to the SSTRs expressed on the surface of NETs and inhibit the secretion of hormones by them, thereby bringing about symptomatic relief to patients. As many as 80% of patients present with metastasis at the time of presentation. Somatostatin analogs, chemotherapy, interferon, tyrosine kinase inhibitors, and peptide receptor radionuclide therapy (PRRT) are the many treatment options available in NET patients [4,5]. It is important to keep gastroenteropancreatic NET as one of the differential diagnoses in our minds when evaluating a patient with chronic non-bloody diarrhea. Due to their rarity, NETs often go undiagnosed for years [6]. However, based on various studies, it has been demonstrated that NETs are not all that rare, and their prevalence has been reported to be as high as 6.6 cases per 100,000 people [7,8].

## Conclusions

Despite being rare tumors, NETs must always be suspected in a patient with complaints of chronic non-bloody diarrhea. Adequate imaging, including 68Ga-DOTANOC PET-CT for metastatic work-up, should be conducted in all patients with a suspicion of NET and somatostatin should be prescribed to all symptomatic patients to avoid other life-threatening consequences.
